# Efficacy and compatibility mechanism of bear bile powder in Shexiang Tongxin dropping pills for acute myocardial infarction treatment

**DOI:** 10.1186/s13020-025-01060-x

**Published:** 2025-01-25

**Authors:** Yu Luo, Fangmin Zhang, Lidan Zhu, Jianfeng Ye, Hong-ye Pan, Xiaoyan Lu, Xiaohui Fan

**Affiliations:** 1 Pharmaceutical Informatics Institute, College of Pharmaceutical Sciences, Zhejiang University, Hangzhou, 310058 China; 2 State Key Laboratory of Chinese Medicine Modernization, Innovation Center of Yangtze River Delt, Zhejiang University, Jiaxing, 314100 China; 3 Zhejiang Conba Pharmaceutical Co., Ltd, Hangzhou, 310051 China; 4 Zhejiang Provincial Key Laboratory of Traditional Chinese Medicine Pharmaceutical Technology, Hangzhou, 310051 China; 5 Jinhua Institute of Zhejiang University, Jinhua, 321299 China

**Keywords:** Bear bile powder, Gut microbiota, Shexiang Tongxin dropping pills, Synergistic compatibility, Acute myocardial infarction, Cardiovascular disease

## Abstract

**Background:**

Bear bile powder (BBP), a unique animal-derived medicine with anti-inflammatory and antioxidant effects, is used in Shexiang Tongxin dropping pills (STDP), which is applied to treat cardiovascular diseases, including acute myocardial infarction (AMI). The efficacy and compatibility mechanisms of action of BBP in STDP against cardiovascular diseases remain unclear. This study aimed to investigate the compatibility effects of BBP in STDP in rats with AMI.

**Methods:**

We investigated the compatibility effects of BBP in STDP in rats with AMI. Non-targeted metabonomics, 16S rRNA analysis, RNA sequencing, and network pharmacology were performed to explore the underlying mechanisms.

**Results:**

The combination of BBP and CF (STDP without BBP) significantly reduced AMI-induced infarction size, pathological alterations of cardiac tissues, and serum lactate dehydrogenase and creatine kinase levels in rats, compared with CF or BBP treatment alone. Gut microbiota and metabonomics results revealed that the combination treatment could upregulate the relative abundance of *Lactobacillus* and downregulate that of *Helicobacter*, *Bilophila*, and *Butyricimonas*, thereby rebalancing the gut microbiota dysbiosis induced by AMI. Consequently, the intestinal metabolite levels of oleoylcholine, glutamylalanine, isokobusone, and hemorphin-4 were altered. However, treatment with CF or BBP alone has a weaker effect on these bacteria. Additionally, the combination treatment induced a 62.34% gene reversion rate compared with 55.56% for BBP and 30.20% for CF treatment alone. Modulation of endothelin 1 and growth factor receptor-bound protein 2 was identified as a key synergistic mechanism underlying the anti-AMI effects of BBP in STDP.

**Conclusion:**

This research provides a scientific explanation of the compatibility of BBP in STDP. Our findings suggested that combination treatment with CF and BBP synergistically attenuates AMI by altering gene expression, gut microbiota, and intestinal metabolite profiles.

**Graphical Abstract:**

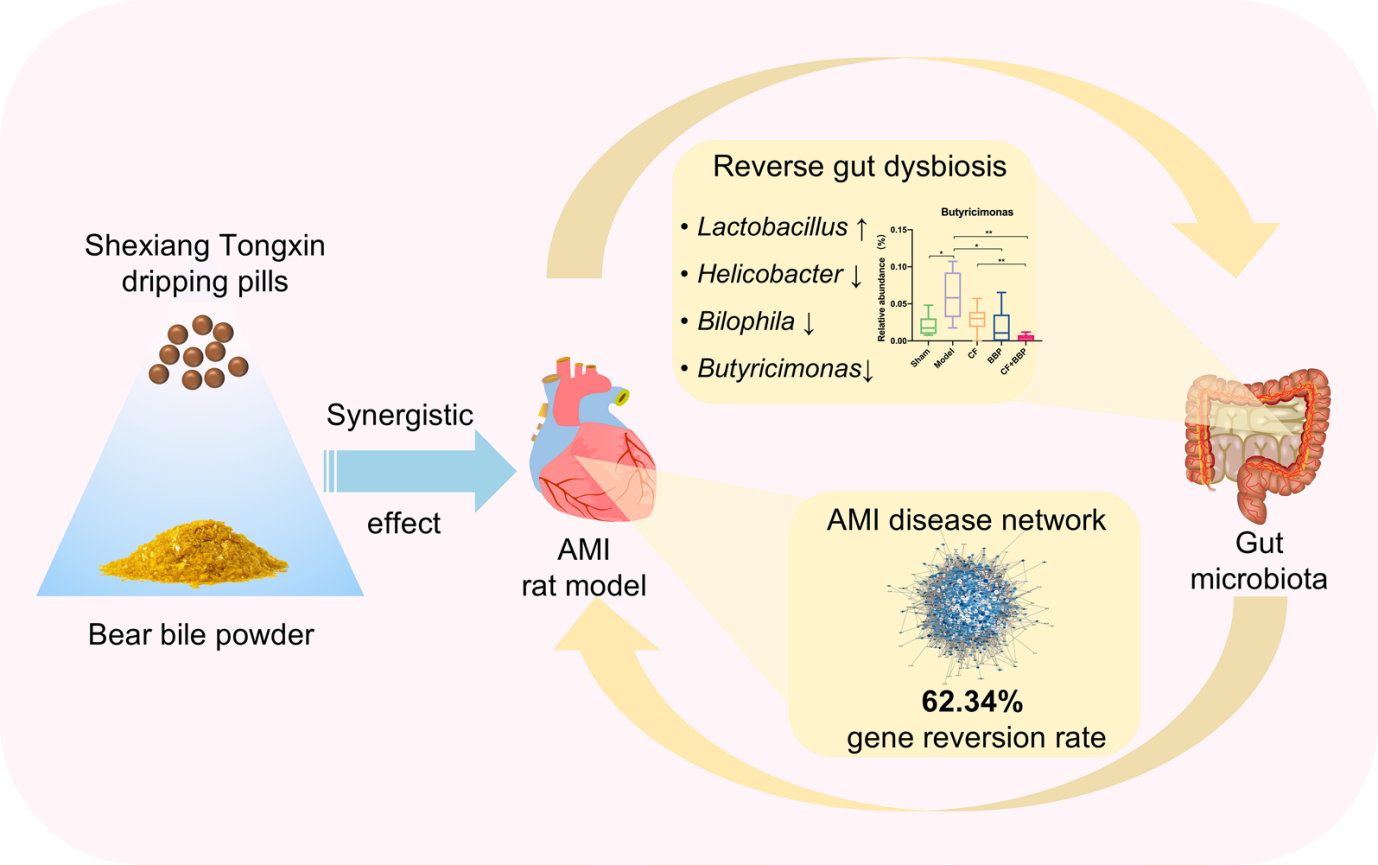

**Supplementary Information:**

The online version contains supplementary material available at 10.1186/s13020-025-01060-x.

## Introduction

Cardiovascular diseases (CVDs) remain the leading cause of death globally, with approximately 17.9 million fatalities annually, according to the World Health Organization [1, 2]. Notably, acute myocardial infarction (AMI) accounts for 46% of these deaths [2]. Current treatment strategies for AMI primarily involve percutaneous coronary intervention, which carries significant complications and high costs [3–5]. Furthermore, pharmacological approaches, such as β-blockers and antiplatelet agents, are limited by their narrow therapeutic scope and potential for serious adverse effects [6]. Thus, developing therapeutic strategies that minimize side effects while providing multi-target efficacy is a critical focus for AMI management. Numerous studies have shown that the onset and progression of AMI are closely associated with inflammation and imbalances in the intestinal microbiota [7–9], highlighting potential therapeutic directions.

Bear bile powder (BBP) is the dried product obtained from *Selenarctos thibetanus Cuvier* or *Ursus arctos* L*.* after gallbladder surgery using the “free-dripping fistula” technique to drain the bile [10, 11]. BBP has a complex chemical composition that includes bile acids, inorganic salts, and trace elements [12]. Among these constituents, bile acids are the most abundant and vital active ingredients in BBP [11]. According to recent pharmacological research, BBP can alleviate vascular occlusion and exert lipid-lowering effects [13]. In clinical practice, BBP is frequently combined with other traditional Chinese medicines (TCMs) to manage conditions such as coronary heart disease and angina pectoris [13]. Moreover, bile acids exhibit a bidirectional relationship with the gut microbiota, and the microbial flora regulate their metabolism, synthesis, and absorption, while their pool and composition influence gut microbiota diversity and homeostasis [14]. BBP is commonly combined with other TCMs to maximize its efficacy.

TCM prescriptions comprise multiple herbs and are characterized by deliberate and essential interactions among herbs possessing distinct functions [15, 16]. The interactions described above include synergism (enhancement of the same effects), adjuncts (enhancement of the effects of other drugs), detoxification (reduction of drug toxicity), antagonism (reduction of the efficacy of other drugs), and repulsion (enhancing toxicity) [16]. Traditional Chinese medicine compatibility (TCMC) is significant in TCM theory, aiming to fundamentally optimize efficacy, minimize adverse effects, and mitigate drug toxicity [15, 17]. BBP is a frequently used ingredient in prescription formulas, with ancient Chinese medicine books and traditional formulas containing as many as 366 formulae featuring BBP [18]. Currently, 153 proprietary Chinese medicines are listed, including BBP, of which 45 are included in the Chinese Pharmacopoeia [19]. Therefore, the compatibility of BBP is essential in numerous herbal medicines.

Shexiang Tongxin dropping pills (STDP) comprise seven types of Chinese medicinal herbs, namely BBP, *Moschus*, *Bufonis venenum*, *Salvia miltiorrhiza* Bge., *Panax ginseng* C.A.Mey., *Calculus bovis artifactus*, and *Borneolum syntheticum* [20, 21]. Recent researches have revealed multiple pharmacological effects of STDP, including anti-inflammatory [22, 23], anti-apoptotic [24], anti-oxidative stress [25], anti-fibrotic [26], and angiogenesis-promoting [23, 27] effects. STDP has been clinically used in treating major CVDs such as heart failure [22, 26], angina pectoris [23], and coronary artery disease [21], owing to its unique prescription ingredients. BBP is a primary component of STDP and distinguishes it from similar proprietary Chinese medicines. Compatibility of BBP is crucial for enhancing the efficacy of STDP. In an atherosclerosis model constructed using apolipoprotein E deficient mice given a diet high in fat, the absence of BBP significantly reduced the anti-oxidative stress and anti-inflammatory effects of STDP [28]. However, the specific mechanism underlying the compatibility of BBP in STDP remains unclear.

Gut microbiota and their metabolites play an important role in AMI [29]. Changes in the community structure of the gut microbiota have been identified in AMI in animal models and human patients, including a decline in the relative abundance of *Lactobacillus* [29, 30]. The gut microbiota creates metabolites such as trimethylamine (TMA) and short-chain fatty acids, which are absorbed into the circulation via the intestinal mucosa [31, 32]. Subsequently, the liver converts TMA to trimethylamine N-oxide (TMAO), which is a significant factor in CVDs [33]. Several investigations have emphasized the critical involvement of intestinal metabolites in AMI onset and progression [34, 35]. Moreover, numerous studies have demonstrated that TCMC yields superior efficacy compared with single-drug administration and is closely associated with the gut microbiota [15]. After antibiotic interference with the gut microbiota, the administration of a mixture of *S. baicalensis* and *S. japonica* had more pronounced anti-hypertensive and nephroprotective effects than the administration of either drug alone [36]. A study on the gut microbiota in the treatment of acute enteritis revealed that the mixture of *S. baicalensis* and *C. chinensis* was more effective when administered together than when administered alone [37]. Therefore, TCMC is crucial in regulating the gut microbiota. This insight may provide a new viewpoint on understanding the TCM theory. Thus, this study intended to elucidate the mechanism underlying the compatibility of BBP in STDP from the viewpoint of gut microbiota and metabolites.

In this study, we employed RNA sequencing and network pharmacology to construct an AMI disease network and explored the compatibility of BBP in STDP. Additionally, we focused on gut microbiota and metabolites using combined 16S rRNA sequencing and metabonomics. Our study provides an in-depth understanding of the compatibility mechanism of BBP in STDP, contributes to the optimization of composition and quality control, and provides a supportive basis for formulation optimization.

## Materials and methods

### Drugs

STDP without BBP is referred to as CF and includes the other six components of STDP, namely, *Moschus*, *Bufonis venenum*, *Salvia miltiorrhiza* Bge., *Panax ginseng* C.A.Mey., *Calculus bovis artifactus*, and *Borneolum syntheticum*. Inner Mongolia Kangenbei Pharmacy Co., Ltd. (Erdos, Inner Mongolia, China) offered CF and BBP (lot no. Y003YC-151110-01). High-performance liquid chromatography (HPLC) analysis of STDP was conducted as previously reported [22]. In this study, CF and BBP were suspended in 1% carboxymethylcellulose sodium (SCRC, Shanghai, China) before intragastric administration. For in vitro experiments, CF (40.00 mg) was ground and dissolved in 1.008 mL of high-glucose or glucose-free Dulbecco’s modified eagle medium (DMEM, Gibco, AL, USA), resulting in a stock solution of 39.68 mg/mL. This suspension was sonicated, filtered through a 0.22 μm membrane for sterilization, aliquoted, and stored at − 20 °C. Prior to use, it was diluted to the desired concentration with a complete or glucose-free culture medium. Similarly, BBP (3.20 mg) was prepared by grinding and dissolving it in 10 mL of high-glucose or glucose-free DMEM to achieve a stock solution of 0.32 mg/mL. The same procedures of sonication, filtration, aliquoting, and storage were followed. The effects of different concentration gradients of CF, BBP, and the combination of CF and BBP on cell viability were then assessed using the cellular models. These analyses enabled the selection of appropriate concentrations for further cell experiments.

### Cell culture and viability assay

Macrophages play a critical role in the inflammatory response, which is closely linked to both the progression and regression of AMI [38–40]. Furthermore, addressing endothelial cell damage has emerged as a pivotal area of research for developing treatments for AMI [41]. Importantly, BBP has demonstrated significant anti-inflammatory properties and protective effects on endothelial cells [10, 25]. Therefore, we selected RAW264.7 cells and human umbilical vein endothelial cells (HUVECs) to investigate the anti-inflammatory and endothelial protective effects of BBP and CF. HUVECs and RAW264.7 cells (both obtained from the Chinese Academy of Sciences Cell Bank) were cultured in DMEM with 10% fetal bovine serum (FBS, Gibco, AL, USA) at 37 °C in a humidified environment with 5% CO_2_. In previous experiments, 100 to 400 μg/mL STDP were found to be non-toxic to RAW264.7 cells, and 200 μg/mL STDP was shown to inhibit lipopolysaccharides (LPS) induced nitric oxide (NO) release from RAW264.7 cells by more than 60% [42]. Based on these results, this study investigated the concentration gradient of STDP (i.e., CF + BBP) on both RAW264.7 cells and HUVECs in the dose range of 100 to 800 μg/mL. Additionally, the concentration gradients for CF and BBP were established based on their respective ratios in the STDP formulation. The effects of varying concentration gradients of CF (198.4, 396.8, and 793.6 μg/mL) and BBP (1.6, 3.2, and 6.4 μg/mL) on cell viability were assessed in HUVECs, while CF (99.2, 198.4, and 396.8 μg/mL) and BBP (0.8, 1.6, and 3.2 μg/mL) were evaluated in RAW264.7 cells. This assessment enabled the identification of optimal concentrations for subsequent experimental applications. The viability of RAW264.7 cells was assessed using the cell counting kit-8 (CCK-8) assay (Yeasen, Shanghai, China) following the manufacturer’s protocol. Following different therapies, after adding 10 µL of CCK-8 to the well, the plate was incubated for 2 h at 37 °C. At 450 nm, absorbance was determined with a spark multimode microplate reader (Tecan, Mannedorf, Switzerland). The viability of HUVECs was evaluated using the methylthiazolyldiphenyl-tetrazolium bromide (MTT) assay. MTT (0.5 mg/mL) was added to each well, and the cells were incubated for 4 h. At the end of the incubation, the supernatant was carefully discarded, and 100 µL of dimethyl sulfoxide was added to each well. Absorbance was measured at 580 nm using a spark multimode microplate reader (Tecan, Mannedorf, Switzerland).

### Detection of NO inhibition rate

Cell passages were carried out utilizing 0.25% trypsin-ethylenediaminetetraacetic acid (trypsin–EDTA, Gibco, AL, USA) when RAW264.7 cells attained confluency of 80–90%. Subsequently, RAW264.7 cells were counted and seeded into 96-well plates, with 2 × 10^4^ cells per well. Following a 24-h incubation period at 37 °C, CF (99.2, 198.4, and 396.8 μg/mL) and BBP (0.8, 1.6, and 3.2 μg/mL) were incorporated into the wells simultaneously with LPS (200 ng/mL). Next, distilled water instead of drugs was incorporated with the LPS in the control wells. After 24 h of incubation at 37 °C, NO was measured using the Griess reagent system kit (Beyotime, Shanghai, China) according to the guidelines supplied by the manufacturer. The absorbance was measured at 540 nm using a Spark multimode microplate reader (Tecan, Mannedorf, Switzerland), and the NO inhibition rate was subsequently calculated according to Eq. 1.

1 $$Inhibition\;rate\;of\;NO\;production\left( \% \right) = \frac{{Absorbance_{the\;model\;group} - Absorbance_{the\;treatment\;group} }}{{Absorbance_{the\;model\;group} - Absorbance_{the\;control \;group} }}$$

### Oxygen and glucose deprivation (OGD) injury model in vitro

HUVECs were used to construct an OGD injury model. When the HUVECs were 80–90% confluent, cell passages were carried out using 0.25% trypsin–EDTA. Subsequently, HUVECs were counted and placed in 96-well plates, with 8 × 10^3^ cells per well as a seed density. HUVECs were cultured in glucose-free DMEM for 8 h at 37 °C in an anaerobic chamber with 5% CO_2_ and 95% N_2_. CF (198.4, 396.8, and 793.6 μg/mL) and BBP (1.6, 3.2, and 6.4 μg/mL) were incubated with cells during OGD injury. After 8 h of hypoxia, the MTT assay was used to determine the cell viability.

### Combination index (CI) calculation

The CI was calculated using the Bliss independence model [43]. The control group is assumed to have a 100% treatment effect compared with the model group, and all other treatment groups are treated as percentages compared with the control group. The combined effect of two drugs can be quantified as ${E}_{CF+BBP}$ (0 ≤${E}_{CF+BBP}$  ≤ 1), and the general formula for probabilistic independence (Eq. (2)) represents the expected additive effect. Specifically, when CI (Eq. (3)) is less than 1, it implies that the two have a synergistic impact.

2 $$E_{CF} + E_{BBP} \left( {1 - E_{CF} } \right) = E_{CF} + E_{BBP} - E_{CF} E_{BBP} \left( {0 \le E_{CF} \le 1, 0 \le E_{BBP} \le 1} \right)$$

3 $${\text{CI}} = \frac{{E_{CF} + E_{BBP} - E_{CF} E_{BBP} }}{{E_{CF + BBP} }} < 1$$

### Animal model and drug administration

The supplier of the male Sprague–Dawley rats (6–8 weeks old; 220–240 g) was Shanghai Slac Lab Animal Technology Co. Ltd. (Shanghai, China). The rats were kept in climate-controlled housing with a 12-h light–dark cycle, consistent temperature (24 ± 1 °C), and relative humidity (55% ± 10%). Food and water were freely available to the rats. All procedures were approved by the Institutional Animal Care and Use Committee of the Zhejiang University School of Medicine, under reference number ZJU20230308.

Following 3 days of acclimation, the rats were split into 5 groups at random, namely sham, model, CF (64.58 mg/kg), BBP (0.52 mg/kg), and CF (64.58 mg/kg) + BBP (0.52 mg/kg). A total of 105 rats were used in this study, with each group consisting of 21 rats. Specifically, in each group, six rats were used for serum biochemical measurement, six for 2,3,5-triphenyltetrazolium chloride (TTC) staining, three for histopathological analysis, three for RNA sequencing, and three for Western blot analysis. According to the calculation of human and rat body surface area conversion value 6.2, the dosages of the above three groups were equivalent to three times the clinical equivalent dosages of CF and BBP in STDP [44, 45]. The gavage volume for the rats was 1 mL/100 g, and the rats received the gavage for 8 days based on their body weights. On the eighth day, after being anesthetized by an intraperitoneal injection of 1% sodium pentobarbital (Sigma, MO, USA), they underwent surgery to establish an AMI model through left anterior descending ligation [46]. The rats were given drugs for 2 days after the operation and sacrificed on the 11th day.

### Serum biochemical measurement

Before being analyzed, serum samples were obtained by centrifuging blood samples for 10 min at 4000 rpm and kept at − 80 °C. The levels of lactate dehydrogenase (LDH) and creatine kinase (CK) in serum were measured using a Cobas C311 automatic biochemical analysis system (Roche Diagnostics, Rotkreuz, Switzerland).

### Determination of myocardial infarct size

After the rat hearts were obtained, six were randomly selected from each group and kept in a refrigerator for 30 min at − 20 °C. The area below the cardiac ligature was divided into five 2 mm-thick slices, and the slices were placed in six-well plates. Subsequently, each well received 2–3 mL of 2% TTC solution. The wells were stained in a light-protected environment for 15 min at 37 °C and turned over with forceps every 5 min to make the staining uniform. Next, the stained hearts were kept in 4% formalin overnight, and all of the sections were photographed the following day. Pale-white areas show infarcted tissues, whereas red parts show non-infarcted tissues. The ratio of the infarcted area to the total heart area was used to quantify the size of the myocardial infarct. The infarcted regions were measured morphometrically using an image analysis system (ImageJ version 1.35e).

### Echocardiography

Transthoracic echocardiography was employed to assess cardiac function and ventricular dimensions of the rat hearts using a VINNO D6 VET ultrasound system (Vinno Technology Ltd., Suzhou, China, equipped with a probe). Measurements were taken 3 days after AMI to provide endpoint data. The rats were anesthetized using isoflurane inhalation (1.5%–2%). The thoracic fur was meticulously removed, and the rats were securely positioned on the operating platform. Echocardiographic recordings of all rat hearts were made at the level of the papillary muscle in both 2D and M-mode. Subsequently, left ventricular ejection fraction (LVEF) and left ventricular fractional shortening (LVFS) were calculated using the VINNO ultrasound system.

### Histopathological analysis

The heart tissues were frozen for 30 min at − 20 °C, followed by scalpel smoothing, repeated ethanol gradient dehydration, and paraffin embedding. After staining tissue slices with hematoxylin and eosin (H&E), they were dehydrated using ethanol and xylene. An optical microscope (Olympus IX53, Tokyo, Japan) was used to view the histopathological changes.

### RNA sequencing

Three cardiac samples from rats in each group were randomly selected for RNA sequencing. Total RNA from the cardiac tissue was extracted by TRIzol reagent (Invitrogen, MA, USA) after 72 h of AMI modeling. RNA purity and quality were examined using an Agilent 2100 bioanalyzer. Subsequently, the cDNA library was constructed using an Illumina NEBNext® UltraTM RNA library prep kit (NEB, MA, USA) following the manufacturer’s instructions. Samples were sequenced using the Illumina NovaSeq platform (Novogene, Beijing, China). Sequencing fragments were converted into sequence data (reads) using CASAVA after the library was qualified. Hisat2 v2.0.5 and featureCounts v1.5.0 were employed to analyze the raw sequencing data to calculate the FPKM (fragments per kilobase of transcript per million fragments mapped) value of each gene. Finally, differentially expressed genes between the two groups were analyzed and *P* value and fold change were calculated by DESeq2.

### Construction and analysis of AMI disease network

An AMI network was built by combining the transcriptomics data with genes linked to CVD, based on our previously established CVD database CHD@ZJU3.0 (http://tcm.zju.edu.cn/chd/) [47]. The network was visualized by Cytoscape V3.9.1 and STRING (https://string-db.org/). The effects of the combination of CF and BBP on the AMI disease network were identified using the efficiency of recovery regulation (EoR; Eq. (4)) and network topology and transcriptomics-based approach (NTRA), as described in our previously published studies [48–50]. EoR was particularly used to quantify the restoration of each gene expression in the AMI disease network after drug administration, with 100% indicating complete recovery. The NTRA rank was utilized to define key genes in the AMI disease and pharmaceutical effect networks from the information of transcriptomics and network topology. In the EoR analysis of the transcriptome data, pathway enrichment analysis was performed on important genes using Metascape (https://metascape.org/gp/index.html).

4 $${\text{EoR}} = 100{\text{\% }} - \left| {100{\text{\% }} - \frac{{{\text{Foldchange}}\left( {\frac{BBP}{{Model}}} \right)}}{{{\text{Foldchange}}\left( {\frac{Sham}{{Model}}} \right)}}} \right|$$

### 16S rRNA library preparation and sequencing

After establishing the AMI model for 3 days, 6 snap-frozen cecal content samples were randomly selected from the rats in each group. The MagPure Soil DNA LQ kit (Magen, Guangzhou, China) was used to extract total microbial genomic RNA from the cecal content samples. The purity and quality of the RNA were examined using agarose gel electrophoresis and NanoDrop2000. Tks Gflex DNA Polymerase (Takara, Tokyo, Japan) was used to amplify the V3 and V4 hypervariable regions of prokaryotic 16S rRNA for taxonomic analysis. Following detection by electrophoresis, RNA was isolated using magnetic beads, measured using a Qubit 2.0 fluorometer, and mixed in equal amounts for 16S rRNA sequencing. Sequencing was performed by Shanghai Ouyi Biomedical Technology Co., Ltd.

### Non-targeted metabonomics analysis

A solution of 600 μL precooled methanol–water (V: V = 4:1) containing 4 μg/mL of L-2-phenylalanine was used to treat each 60 mg cecal content sample. The mixture was ground with two small steel beads for 2 min at 60 Hz, centrifuged at 12,000 rpm for 10 min at 4 °C after being ultrasonicated for 10 min in an ice water bath, and incubated for 30 min at − 40 °C. Following collection and filtration via a 0.22-μm syringe filter, the supernatant was analyzed using liquid chromatography-mass spectrometry (LC–MS). Equal amounts of extract from each sample were combined to create quality control samples.

LC–MS analysis was performed by an ultra-performance liquid chromatography-tandem QE high-resolution mass spectrometer (ACQUITY UPLC I-Class plus, Waters, MA, USA). Reversed-phase separation was performed on an ACQUITY UPLC T3 column (100 × 2.1 mm, 1.8 µm; Waters, MA, USA). The temperature was set to 45 °C, with a flow rate of 0.35 mL/min. Solvents A (water and 0.1% formic acid) and B (acetonitrile) consisted of the mobile phase. The gradient elution parameters are shown in Table S1. The mass spectrometry system used an electrospray ion source, with sample signal acquired using performed positive and negative ion scanning modes; the mass spectrometry settings are listed in Table S2.

Progenesis QI v3.0 was used to pre-process the raw data files. Compounds were identified by the human metabolome database, METLIN databases, LipidMaps v2.3, and EMDB2.0 for metabolite characterization based on isotope analyses, secondary fragmentation, and accurate mass. The connection between metabolite expression and sample grouping was determined by partial least squares discriminant analysis (PLS-DA). Differentially expressed metabolites between the model group and CF or BBP or CF and BBP combined group were analyzed by *T* test, with *P* < 0.05, |log_2_ (Fold Change)|> 0.2, and compound qualitative result score (Score) ≥ 38.1 set as screening conditions.

The sample pre-processing operations for gas chromatography–mass spectrometry (GC–MS) were the same as those for LC–MS. The supernatant was centrifugally concentrated and dried after centrifuging for 10 min, a solution of methoxyamine hydrochloride pyridine solution was added and then shaken at 37 °C for 60 min to perform the oximation reaction. Subsequently, bis(trimethylsilyl)trifluoroacetamide derivatization reagent, n-hexane, and 10 internal standards (methyl octanoate, methyl nonanoate, methyl caprate, methyl dodecanoate/methyl laurate, methyl myristate/methyl myristate, methyl hexadecanoate/methyl palmitate, methyl octadecanoate/methyl stearate, methyl eicosanoate/methyl arachidonate, methyl docosanoate/methyl santalic acid, methyl tetradecanoate/methyl lignocarbonate) were added. The solution was heated to 70 °C for 60 min and being held at room temperature for 30 min for GC–MS analysis.

GC separation was performed on a 30-m DB-5MS column (0.25-mm i.d. × 0.25-μm film thickness; J&W Scientific, CA, USA). The injection volume was 1 μL at 260 °C without splitting, with a solvent delay of 6.2 min, and high-purity nitrogen (1.0 mL/min) carrier gas. Mass spectrometry conditions included a 230 °C electron impact ionization source, a 150 °C quadrupole temperature, and a 70-eV electron energy. The full-scan mode, which covers a mass range of m/z 50 to 500, was set as scanning mode. MS-DIAL was used to pre-process the raw GC–MS data. The output included sample information, substance peak names, a raw data matrix of retention time, *m/z*, and mass spectral response intensity (peak area). Compound identification for GC–MS was performed using the LUG database (untargeted database of GC–MS of Lumingbio) independently developed by Lumingbio.

### Real-time quantitative reverse transcription polymerase chain reaction assay (RT-qPCR)

RT-qPCR was used to validate the sequencing data on a Bio-Rad CFX96 Touch™ real-time PCR detection system (Bio-Rad, CA, USA). A NanoDrop 2000 was used to determine the RNA concentration extracted from each sample. Samples from each group were diluted to the same concentration using RNase-free water (Beyotime, Shanghai, China). Mixed RNA samples were reverse transcripted using the HiFiScript cDNA synthesis kit (CoWin, Jiangsu, China). Hieff UNICON® universal blue qPCR SYBR green master mix (Yeasen, Shanghai, China) and specific primers (Sangon, Shanghai, China) were used to quantify the gene expression on a CFX-Touch™ 96 real-time PCR system (Bio-Rad, CA, USA). Table S3 lists the exact primer sequences. The relative gene expression was assessed using the 2^−ΔΔCt^ technique, with *Gapdh* as the internal control. The experiment was repeated thrice.

### Western blot analysis

Total proteins were isolated from the cardiac tissue. BCA protein assay kit (Thermo Fisher Scientific, MA, USA) was used to detect the protein concentrations. Protein samples were separated on a 10% sodium dodecyl sulfate–polyacrylamide gel and electrophoretically transferred to polyvinylidene fluoride membranes (Merck Millipore, Hesse, Germany). To block the nonspecific binding sites, the membranes were incubated with a solution of 5% nonfat milk and Tris-buffered saline with Tween-20 (TBST). Subsequently, the membranes were incubated at 4 °C overnight with anti-endothelin 1 (EDN1), anti-growth factor receptor-bound protein 2 (GRB2), and anti-glyceraldehyde 3-phosphate dehydrogenase (GAPDH) antibodies. After rinsing, horseradish peroxidase-labeled goat anti-rabbit IgG was used to incubate membranes at room temperature for 1 h. After washing with TBST, an enhanced chemiluminescent substrate reagent (Merck Millipore, Hesse, Germany) and a ChemiDoc™ imaging system (Bio-Rad, CA, USA) were used to visualize the blot. Image Lab software 6.1 was used to analyze the relative protein levels, and the experiment was repeated thrice.

### Statistical analysis

Data presented as mean ± standard error of the mean (SEM) were analyzed using GraphPad Prism 8.4.0 software. One-way analysis of variance, followed by Dunnett’s post-hoc analysis, was used to examine statistical differences. *P* < 0.05 was defined as statistically significant. OECloud tools (https://cloud.oebiotech.com) were used for 16S rRNA data analysis.

## Results

### BBP combined with CF treatment has anti-inflammatory and anti-hypoxic properties in vitro

**Fig. 1:**
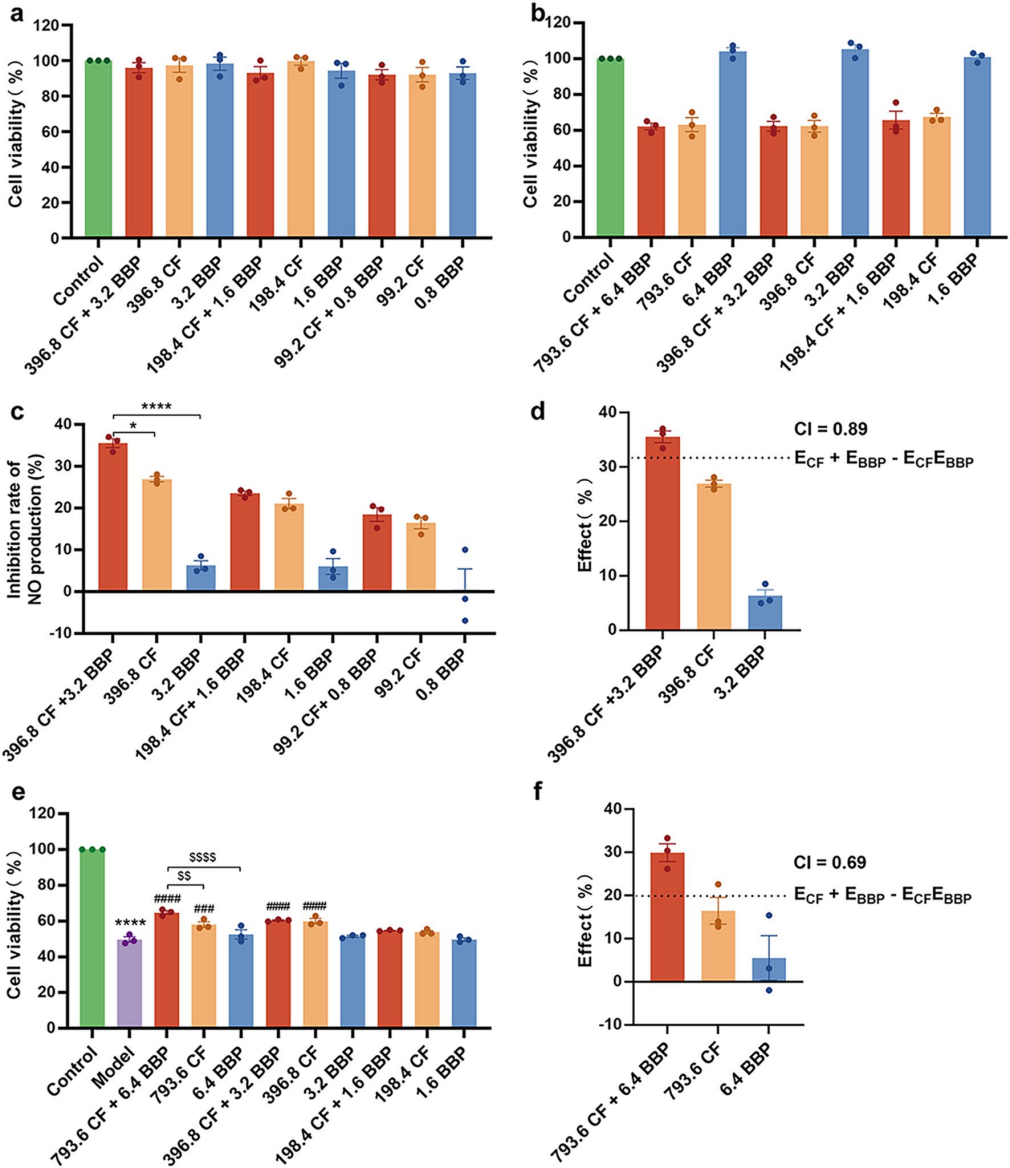
BBP compatibility in STDP has anti-inflammatory and anti-hypoxic injury effects in vitro. **a** RAW264.7 cell viability after CF and BBP treatment. **b** HUVECs cell viability after CF and BBP treatment. **c** NO inhibition by CF and BBP in RAW264.7 cells stimulated by LPS. **d** The CI of the anti-inflammatory activities of CF and BBP. **e** The OGD-injured HUVEC model was treated with CF and BBP. **f** The CI of CF and BBP on the protective effects of OGD-injured HUVECs. Data are presented as mean ± SEM; *n* = 3 per group. *P < 0.05, ***P < 0.001, ****P < 0.0001 vs. the control group, ^###^*P* < 0.001, ^####^*P* < 0.0001 vs. the Model group, ^$$^*P* < 0.001, ^$$$$^*P* < 0.0001 vs. the drug delivery group. The numbers in the graph represent drug concentrations (μg/mL). CF, STDP without BBP; BBP, bear bile powder; CF + BBP, the combination of CF and BBP

Different concentrations of BBP and CF were applied to RAW264.7 cells and HUVECs to select non-toxic concentrations for efficacy evaluation. As shown in Fig. 1a, CF (99.2, 198.4, and 396.8 μg/mL) and BBP (0.8, 1.6, and 3.2 μg/mL) did not affect viability in RAW264.7 cells. Conversely, when compared to the control group, the viability of HUVECs was considerably decreased by CF (198.4, 396.8, and 793.6 μg/mL) therapy (Fig. 1b), indicating a slight toxicity of CF toward HUVECs. Previous studies have suggested that *Bufonis venenum* contains toxic components, and its extracts inhibit the proliferation of HUVECs [51]. Therefore, the *Bufonis venenum* present in CF may have relevant cytotoxic effects.

We constructed an inflammatory response model using LPS-induced RAW264.7 cells and assessed the inhibitory effect of BBP compatibility in STDP on NO production. The inhibition rate of NO was significantly higher when treated with a combination of CF (396.8 μg/mL) and BBP (3.2 μg/mL) than individual treatments (Fig. 1c). The NO inhibition rate following the combination of CF (198.4 and 99.2 μg/mL) and BBP (1.6 and 0.8 μg/mL) was higher than when CF and BBP were administered individually at the corresponding concentrations. However, the difference was not statistically significant. As the CI = 0.89 < 1 (Fig. 1d), it suggests the synergistic anti-inflammatory effect of CF and BBP at this concentration.

An OGD-injured HUVEC model was established to explore the anti-hypoxic effects of BBP compatibility in STDP. Treatment with CF at 793.6 μg/mL combined with BBP at 6.4 μg/mL caused a significantly higher cell viability than that in the model group. Similarly, this combination caused higher cell viability than treatment with CF or BBP alone (Fig. 1e). Additionally, CI calculations revealed a value of 0.69 (< 1), suggesting that CF and BBP have synergistic antioxidant effects against hypoxic injury in vascular endothelial cells at this concentration (Fig. 1f). In combination with the analysis of Fig. 1b, it was also found that CF was toxic to HUVECs at a dosage of 793.6 μg/mL; however, CF at this concentration had a protective effect on HUVECs under OGD injury, indicating that, as with many TCMs, CF exerts different effects in normal and pathological conditions.

### BBP combined with CF treatment alleviated myocardial infarction area and pathological damage while enhancing cardiac function in rats with AMI

**Fig. 2:**
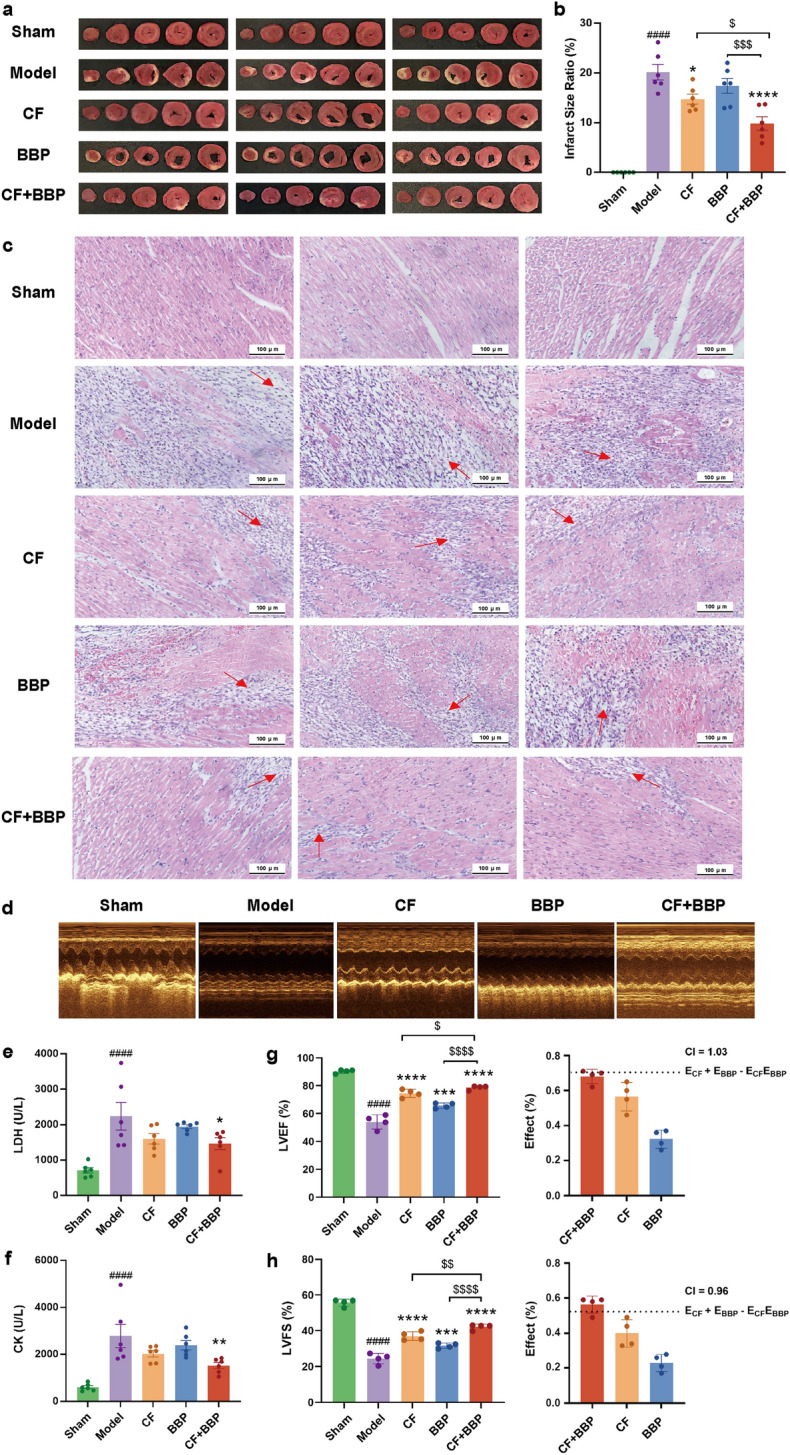
Combining CF and BBP decreased the infarct size and cardiac morphological change.** a** TTC staining of heart tissue in rats with AMI after the treatment with CF, BBP and CF + BBP, *n* = 6. **b** Infarct size of cardiac tissue in rats with AMI after the treatment with CF, BBP and CF + BBP, *n* = 6. **c** H&E staining of cardiac section in rats with AMI after the treatment with CF BBP and CF + BBP, *n* = 3. **d** M-mode echocardiograms showing at 3 days after AMI. **e** Serum LDH levels in rats with AMI, *n* = 6. **f** Serum CK levels in rats with AMI, *n* = 6. **g** Statistics of LVEF and CI in rats with AMI after treatment with CF, BBP, and CF + BBP, *n* = 4. **h** Statistics of LVFS and CI in rats with AMI after treatment with CF, BBP, and CF + BBP, *n* = 4. Data are presented as mean ± SEM. ^####^*P* < 0.0001 vs. the control group, *P < 0.05, **P < 0.01, ****P < 0.0001 vs. the Model group, ^$^*P* < 0.05, ^$$$^*P* < 0.001 vs. the drug delivery group. CF, separated prescription (STDP without BBP); BBP, bear bile powder; CF + BBP, the combination of CF and BBP

To assess the impact of BBP compatibility in STDP on AMI, an AMI model was established after 8 days of continuous gavage administration of CF, BBP, and CF + BBP. TTC and H&E staining were performed on rat heart tissues. In contrast to the model group, which displayed a pale hue in the infarcted hemisphere with an average infarct area ratio of 20.16%, the sham group had a red color in the heart tissues without any infarction. The combination of CF and BBP greatly reduced the extent of infarction, with the ratio decreasing to 9.85%. This therapeutic effect was superior to that of CF or BBP alone (Fig. 2a, b). Similarly, the results of H&E staining indicated that the combined effects of CF and BBP superior to the results of either therapy alone (Fig. 2c). Notably, LDH and CK activities and interleukin-6 (IL-6) levels in the serum of rats with AMI reflected similar outcomes. The combination of CF and BBP significantly reduced the increased IL-6, LDH, and CK levels caused by myocardial infarction; however, CF and BBP alone did not exhibit such pronounced effects on LDH and CK levels (Fig. 2e, f and Fig. S1). Furthermore, we evaluated the effects of combined CF and BBP on cardiac function in rats with AMI by analyzing crucial echocardiographic parameters. Rats with AMI exhibited a significant reduction in LVEF and LVFS compared with the sham group (Fig. 2d, g, h). In contrast, treatment with either CF or BBP alone, as well as the combination of CF and BBP, led to significant restoration of LVEF and LVFS in rats with AMI, compared with the model group (Fig. 2d, g, h). Moreover, the combination treatment of CF and BBP was notably more effective than either treatment alone in enhancing cardiac LVEF and LVFS. Additionally, CI calculations yielded a value of 0.96 (< 1), indicating a synergistic effect of CF and BBP in restoring LVFS (Fig. 2h). These findings suggest that the combination of CF and BBP obviously attenuates the infarct size, mitigates morphological changes, and enhances cardiac function in rats with AMI.

### BBP combined with CF treatment reversed the disturbed gene expression in AMI disease network

**Fig. 3:**
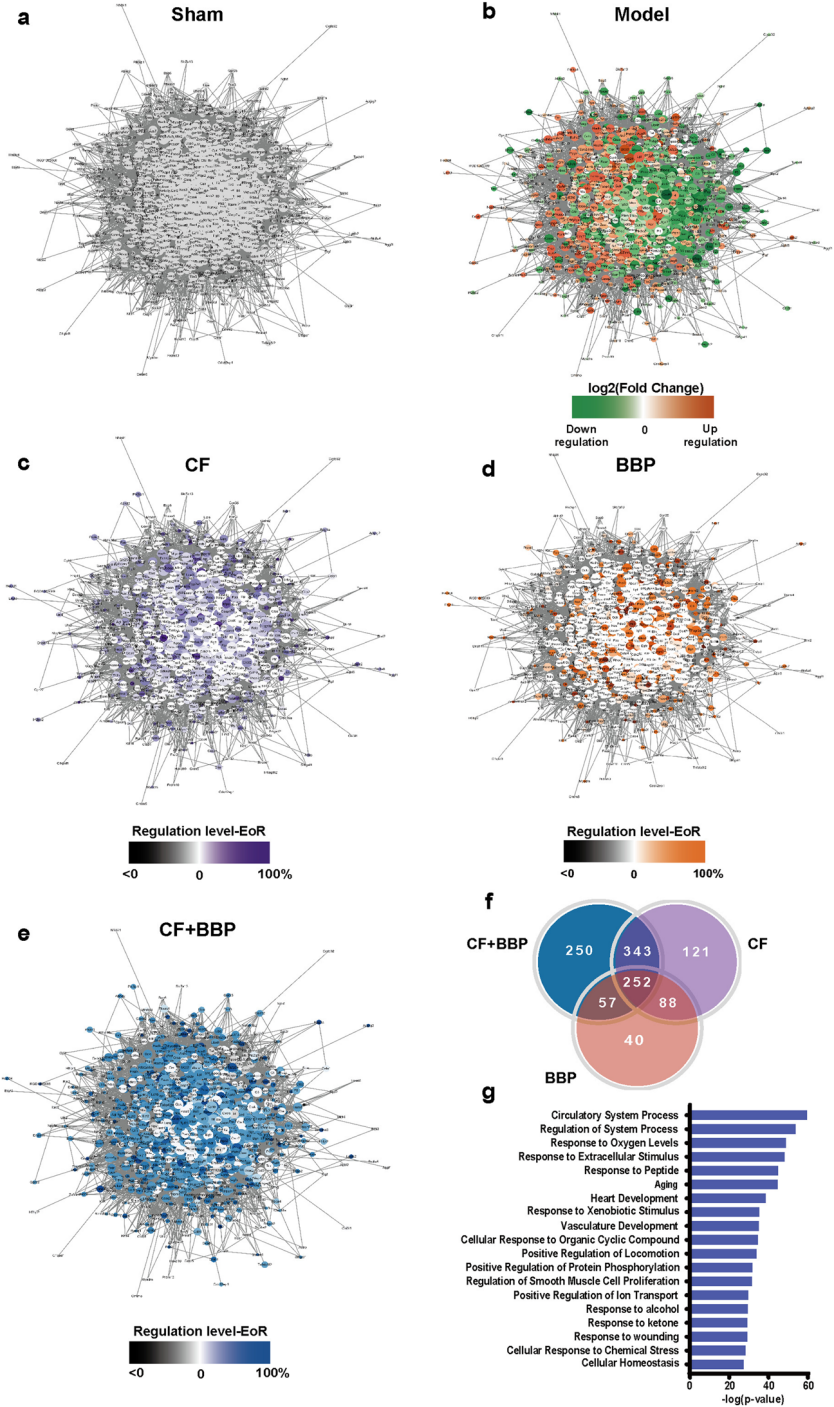
Effect of the combination of CF and BBP on AMI disease networks. **a** The sham group. The node size represents the NTRA ranking; the higher the ranking, the larger the node. **b** The model group. Green indicates downregulation of gene expression following AMI, while red indicates upregulation following AMI; darker colors correspond to more pronounced up- and down-regulation of the gene. **c** The CF group. **d** The BBP group. **e** The combination of CF and BBP group. **f** Venn plot of recovery regulated genes (EoR > 0) for each drug delivery group. **g** The top 19 pathways obtained after GO terms enrichment analysis. The purple, orange and blue colors represent the degree of gene up-regulation (EoR) after drug administration compared to the Model; the darker the color, the more extensive the regulatory recovery, and the black color represents no recovery regulation of the gene. CF, separated prescription (STDP without BBP); BBP, bear bile powder; CF + BBP, the combination of CF and BBP

In order to investigate the mechanism of action of the combination of CF and BBP against AMI, we used transcriptomics and network pharmacology to create a network related to AMI. By comparing 1641 genes associated with CVDs from our previous study [47] with the 32,545 genes obtained from transcriptome sequencing, we identified 1539 shared genes. Furthermore, an AMI disease network containing 1447 nodes and 44,879 connections was constructed using protein–protein interactions (PPI) data from the STRING database (Fig. 3a). In the network, node colors reflect gene expression changes, with red indicating upregulation and green indicating downregulation through AMI modeling based on log_2_ (Fold Change) comparing the sham and model groups (Fig. 3b). The EoR value indicates the impact of CF and BBP compatibility on disease-related genes. The colored network in Fig. 3b indicates gene expression disorders in the cardiac tissues of rats with AMI and impaired cardiac function. However, when co-administered with CF and BBP, 62.34% (902 out of 1447) of the genes showed recovery regulation. In contrast, treatment with CF alone (Fig. 3c) regulated 55.56% (804 out of 1447) of the genes, and treatment with BBP alone (Fig. 3d) regulated only 30.20% (437 out of 1447) of the genes. Therefore, CF and BBP exerted synergistic effects in rebalancing the AMI disease network (Fig. 3e). Subsequently, genes showing recovery regulation (EoR > 0) were analyzed within each group. The combination of CF and BBP specifically recovery-regulated 250 genes compared with the CF or BBP alone administration groups (Fig. 3f), suggesting potential novel targets caused by the synergy between CF and BBP.

**Fig. 4:**
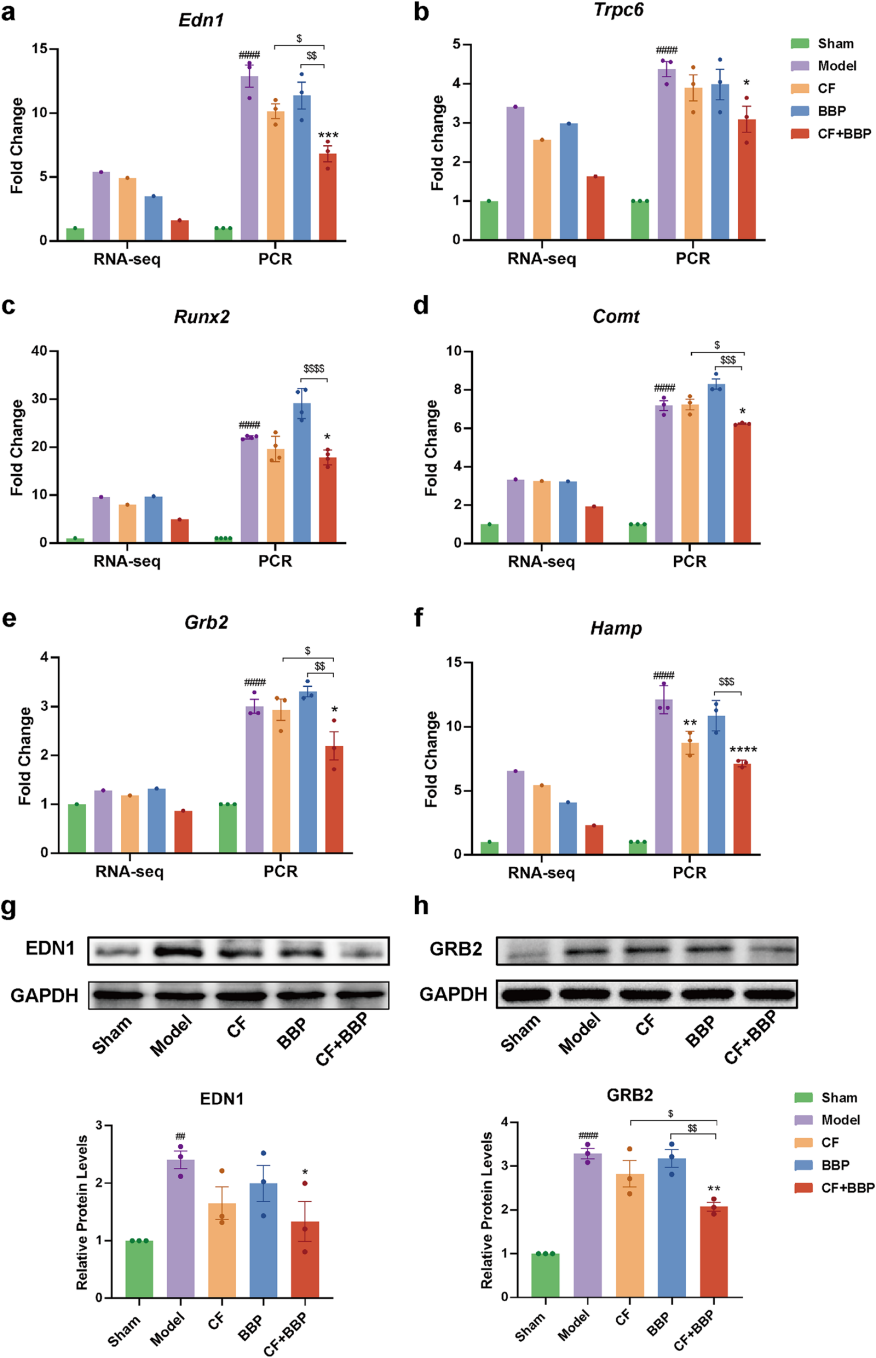
Expression of key genes and proteins in rat heart tissue. **a**–**f** The gene expression levels of *Edn1*, *Grb2*, *Trpc6*, *Runx2*, *Comt*, and *Hamp* were identified using transcriptome sequencing and qRT-PCR. *Gapdh* served as the internal reference, *n* = 3. **g** Expression of EDN1 proteins in rat heart tissue, *n* = 3. **h** Expression of GRB2 proteins in rat heart tissue, *n* = 3. Data are presented as mean ± SEM. ^##^*P* < 0.01, ^####^*P* < 0.0001 vs. the Sham group, *P < 0.05, **P < 0.01, ***P < 0.001, ****P < 0.0001 vs. the Model group, ^$^*P* < 0.05, ^$$^*P* < 0.01, ^$$$^*P* < 0.001 vs. the drug delivery group. CF, separated prescription (STDP without BBP); BBP, bear bile powder; CF + BBP, the combination of CF and BBP

Moreover, in the AMI disease network, the screening was used to identify 492 genes for which the gene recovery regulation in the combination group was greater than 0.2 compared with that in the CF or BBP alone administration group. Briefly, there were 492 genes for which the difference between the EoR value in the combination group and that in the CF or BBP alone administration group exceeded 0.2. Following the elimination of duplicated genes between 250 and 492 genes, 537 genes were identified in the AMI disease network. These genes were analyzed for gene ontology (GO) terms enrichment, and Fig. 3g displays the top 19 pathways. Among these pathways, response to extracellular stimulus, circulatory system process, response to oxygen levels, regulation of the system process, heart development, vasculature development, and regulation of smooth muscle cell proliferation were the most significantly impacted. Subsequently, we focused on genes that were evidently affected by these pathways. *Edn1*, which is essential for the control of superoxide and maintenance of normal cardiac contractile function, is significantly affected in the circulatory system process and response to oxygen level pathways [52]. Previous investigations have shown that reduced expression of *Edn1* facilitates cardiac recovery following myocardial infarction, a process closely related to the modulation of vascular smooth muscle cell proliferation and immune response after AMI [53, 54]. Furthermore, increased transcript levels of *Edn1* have been observed during simulated cardiac fibrosis in vitro [55]. The GRB2 (encoded by *Grb2*) and transient receptor potential cation channel subfamily C member 6 (TRPC6, encoded by *Trpc6*) are associated with the response to the extracellular stimulus pathway through the modulation of extracellular signal-regulated kinase 1/2 activity [56, 57]. The Grb2-p38 MAPK pathway is vital to cope with pressure overload-induced cardiac hypertrophy and myocardial fibrosis [58, 59]. Hypertrophic cardiomyopathy and cardiac failure are caused by the overexpression of TRPC6 [60], and the inhibition of its expression ameliorates cardiac fibrosis and myocardial ischemia–reperfusion injury [61, 62]. Runt-related transcription factor 2 (*Runx2*) was significantly affected in the heart development pathway. It is involved in vascular calcification, and its mRNA and protein expressions were found to be upregulated in myocardial infarcted mouse hearts, demonstrating that *Runx2* prevents adverse cardiac remodeling through vascular endothelial cells [63]. Low-activity catechol-O-methyltransferase (*Comt*) prevents myocardial infarction and reduces its risk [64, 65]. The peptide derived from the hepcidin antimicrobial peptide (*Hamp*) is associated with the regulation of systemic process pathways, particularly iron metabolism. In addition, *Hamp* expression is significantly upregulated in cardiac tissues after AMI, suggesting that *Hamp* plays a significant role in AMI [66, 67]. The key genes related to AMI in the enriched pathways were further validated using RT-qPCR. The gene expression levels of *Edn1*, *Grb2*, *Trpc6*, *Runx2*, *Comt*, and *Hamp* were consistent with the transcriptome sequencing results (Fig. 4a–f). The aforementioned results verify the accuracy of the transcriptome sequencing data and indicate that these six genes play key roles in the combination of CF and BBP against AMI.

Studies have indicated that EDN1 can inhibit fibroblast apoptosis by activating the PI3K/AKT pathway [68], and that restraint of the EDN1-induced PI3K/AKT pathway can further alleviate cardiac hypertrophy [69]. GRB2 induces cardiac fibrosis and hypertrophy through the stimulation of the p38 MAPK and JNK pathways [59]. Therefore, EDN1 and GRB2 may play key roles in AMI development. We further verified the alterations in the expression of EDN1 and GRB2. Compared with the sham group, the expression of EDN1 and GRB2 proteins was substantially upregulated in the model group. In the combined CF and BBP treatment group, the protein expression of EDN1 and GRB2 was significantly downregulated as opposed to that in the model group, and the regression trend was more significant than that observed with CF or BBP administration alone (Fig. 4g, h). These results suggest that the modulation of EDN1 and GRB2 is a key mechanism of the synergistic anti-AMI effect when BBP is formulated in STDP.

### BBP combined with CF treatment obviously recovered the gut dysbiosis in AMI rat models

**Fig. 5:**
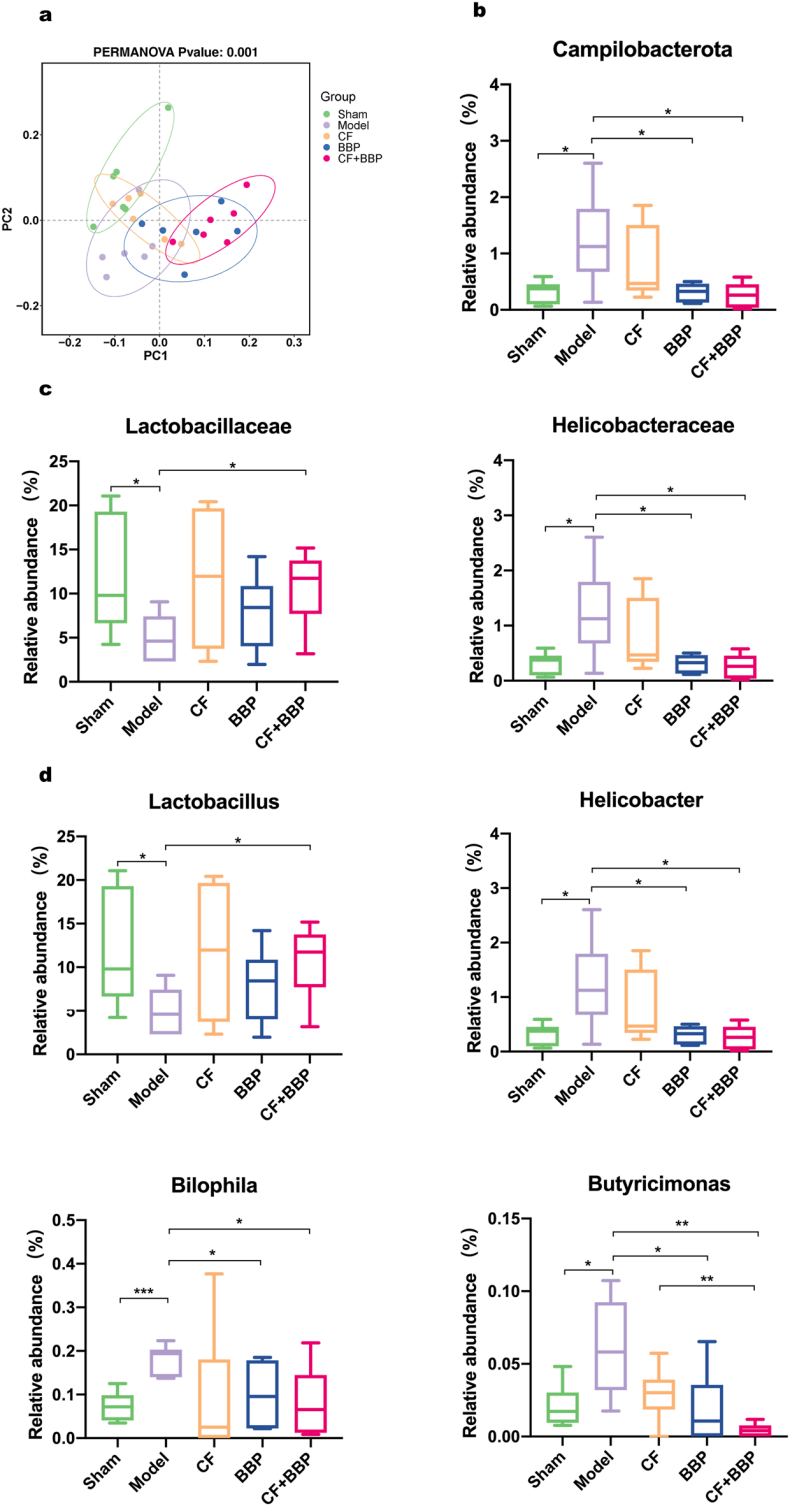
The combination treatment of BBP and CF alleviates gut dysbiosis in AMI rat model. **a** PCoA plots of the gut microbiota in cecal content samples. Gut bacteria that are significantly restored by CF and BBP at the levels of phylum (**b**), family (**c**), and genus (**d**). *n* = 6 per group, ^*^P < 0.05*,* ^**^P < 0.01*,* and ^***^P < 0.001 vs. Model group. CF, separated prescription (STDP without BBP); BBP, bear bile powder; CF + BBP, the combination of CF and BBP

Based on 16S rRNA sequencing, we examined the changes in the gut microbiota of rats after AMI modeling and subsequent drug treatment. Principal coordinate analysis (PCoA), using the unweighted Unifrac metric, revealed that the samples from the group treated with the combined CF and BBP therapy were notably differentiated from those in the model group, and this phenomenon was not observed in the groups that received CF or BBP treatments alone (Fig. 5a). Moreover, at the level of phylum, family, and genus, the combination of BBP and CF significantly restored the relative abundance of unbalanced gut microbiota caused by AMI in rats. At the levels of phylum and family of gut microbiota, the combination of CF and BBP, or BBP alone, restored the relative abundance of *Campilobacterota* (Fig. 5b), *Lactobacillaceae*, and *Helicobacteraceae* in rats with AMI (Fig. 5c). Additionally, the combination of CF and BBP exhibited a more pronounced restorative effect. *Campilobacterota* is a pro-inflammatory bacterium that is known to increase in relative abundance in some inflammatory diseases, such as mouse colitis [70]. Since the inflammatory response is crucial in AMI prognosis and progression [71], the combination of CF and BBP may reduce the inflammatory response after AMI by reversing the relative abundance of pro-inflammatory bacteria like *Campilobacterota.* At the genus level, administration of CF and BBP also restored the relative abundance of *Lactobacillus, Helicobacter*, *Bilophila*, and *Butyricimonas* in rats with AMI (Fig. 5d). *Lactobacillus* can improve cardiac function by improving inflammation and myocardial damage after AMI [72], reducing the levels of tumor necrosis factor-α and lipid peroxidation in myocardial infarction rats [73]. *Helicobacter* can induce the production of inflammatory factors [74, 75] and increase the risk of myocardial infarction [76]. *Bilophila* is positively correlated with TMAO levels in plasma, which has been shown to cause atherosclerosis [77], aggravate heart failure [78], and promote thrombosis [79], vascular inflammation, inflammasome activation [80]. *Butyricimonas*, which helps resist oxidative stress and repair the intestinal mucosal barrier [81], is more abundant in patients with AMI than in healthy individuals [31]. Further details on the gut microbiota composition of the samples in these groups at the levels of phylum, family, and genus are shown in Fig. S1.

**Fig. 6:**
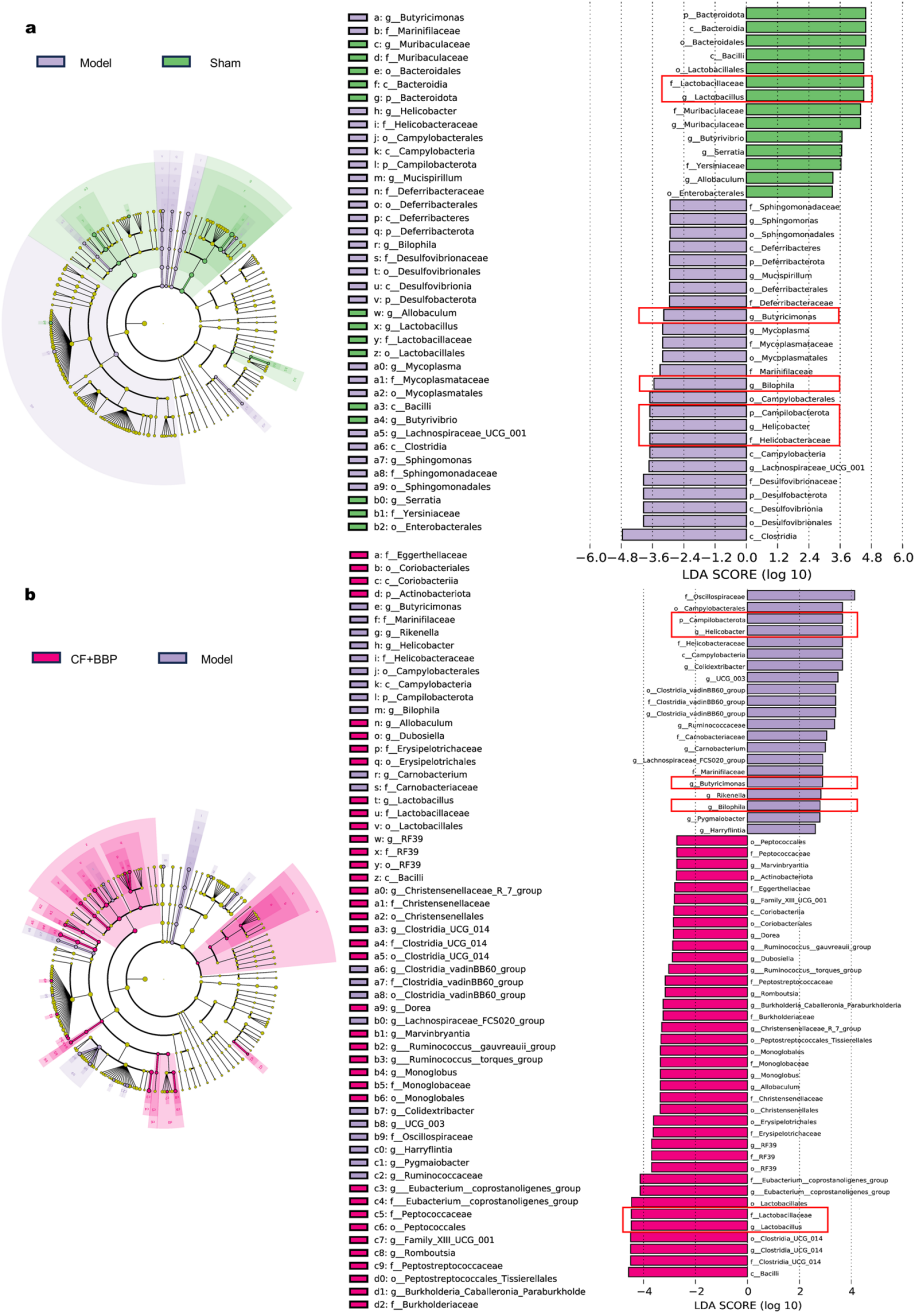
LEfSe of the genus-level designations for the gut microbiota in model, sham, and CF + BBP. **a** Model vs. Sham group. **b** Model vs. BBP + CF group. From the innermost to the outermost rings indicate phylum, class, order, family, and genus, respectively.* n* = 6 per group. CF, separated prescription (STDP without BBP); BBP, bear bile powder; CF + BBP, the combination of CF and BBP

To compare genus levels among these groups, linear discriminant analysis Effect Size (LefSe) analyses were performed with a linear discriminant analysis (LDA) threshold of 2.5 (Fig. 6). The results revealed that *Lactobacillaceae* and *Lactobacillus* were the main bacteria shared by the sham and the combined CF and BBP groups (Fig. 6a). *Campilobacterota*, *Helicobacteriaceae*, *Helicobacter*, *Bilophila,* and *Butyricimonas* were the dominant bacteria that only existed in the model group (Fig. 6b). Depending on the LefSe analysis results, we can conclude that the combination of CF and BBP actively restored the main bacteria in the rat model group, therefore reduced AMI damage severity.

### BBP combined with CF treatment could reduce the disturbances in the intestinal metabolite profile of rats with AMI

**Fig. 7:**
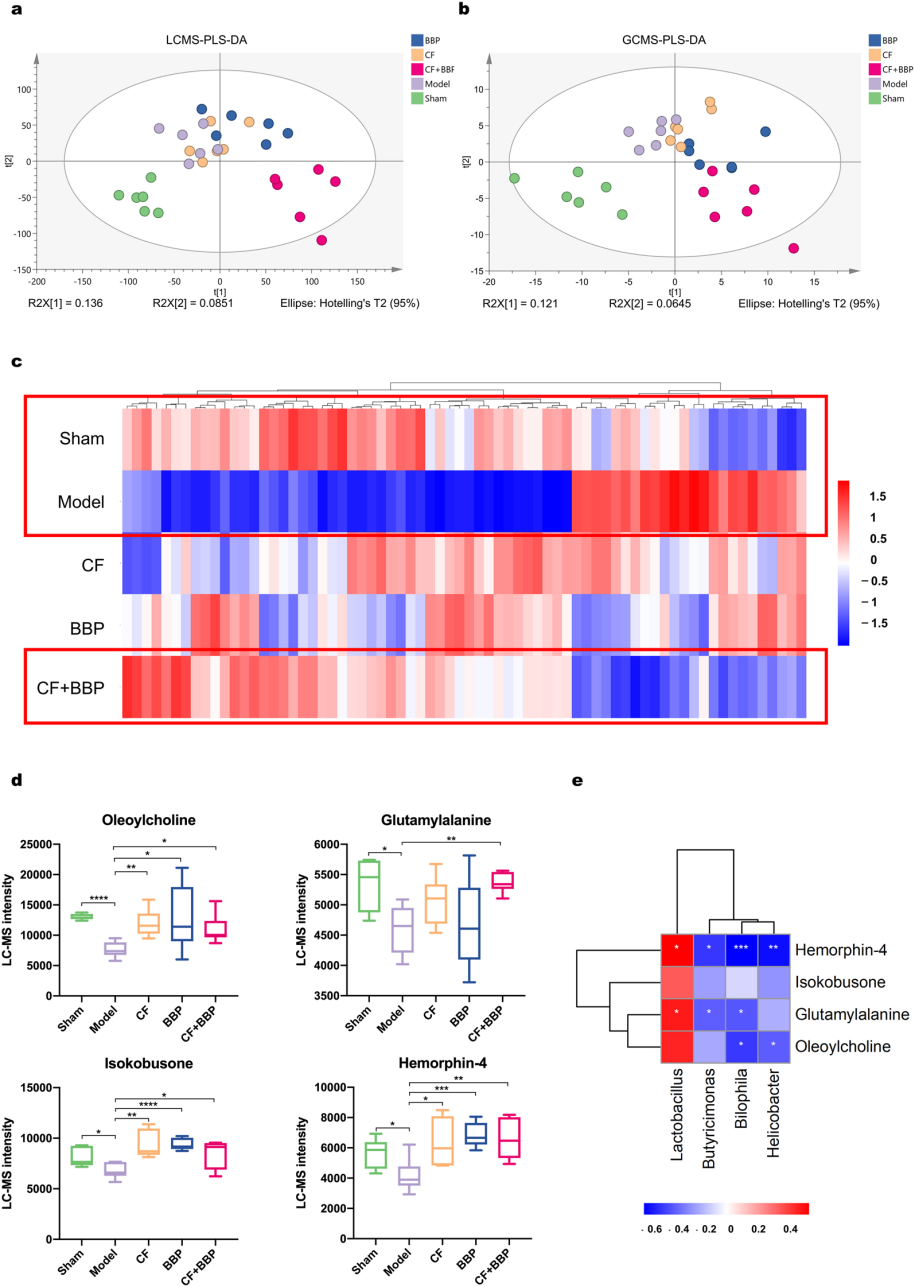
The differences in intestinal metabolites between LC–MS and GC–MS. Scatter plot of PLS-DA model of LC–MS (**a**) and GC–MS (**b**). **c** Hierarchical cluster analysis of the 70 differential metabolites in the intestinal metabolites. **d** Four differential metabolites are closely related to AMI. The different gene expression levels were colored in different colors, with red representing highly expressed genes and blue representing lowly expressed genes. **e** The analysis of Spearman’s correlation heatmap coefficients illustrates the relationship between intestinal metabolites and different abundance bacterial genera.* n* = 6 per group, *P < 0.05, **P < 0.01, and ***P < 0.001 vs. Model group. CF, separated prescription (STDP without BBP); BBP, bear bile powder; CF + BBP, the combination of CF and BBP

Untargeted metabonomics analysis using GC–MS or LC–MS was used to characterize the effects of the combined CF and BBP in AMI rats on the intestinal metabolite profile. PLS-DA was used to analyze the data matrices acquired from LC–MS (Fig. 7a) and GC–MS (Fig. 7b). The PLS-DA plots revealed significant changes in the metabolite profiles between the sham group and the AMI model group. The combination of CF and BBP demonstrated a trajectory moving away from the model group and toward the sham group, suggesting that the combination of CF and BBP brings the intestinal metabolite profile of rats affected by AMI closer to that of rats in the normal group.

Volcano plot analysis was performed to further compare the differential metabolites (Fig. S2). It showed that AMI modeling yielded 793 LC–MS and 36 GC–MS differentially expressed metabolites compared with the control group. Compared with the model group, the CF and BBP combination treatment group showed differential expression of 1,008 LC–MS and 56 GC–MS metabolites. CF alone caused the differential expression of 466 LC–MS and 20 GC–MS metabolites, whereas BBP alone caused the differential expression of 482 LC–MS and 35 GC–MS metabolites. A *T* test analysis of differentially expressed metabolites was performed among different groups, with the screening criteria of *P* < 0.05, |log_2_ (Fold Change)|> 0.2, and a compound identification score ≥ 38.1. This analysis was used to identify 70 differential metabolites that were restored in the combined CF and BBP treatment group compared to the model group. Subsequently, 70 metabolites were plotted as differential metabolite heat maps (Fig. 7c). The heatmap demonstrates that AMI modeling induced disruptions in intestinal metabolites in rats. However, the combination of CF and BBP reversed these changes and exhibited a more significant effect than CF or BBP alone. Based on previous studies, we identified four metabolites that were deeply related to the incidence and progression of AMI, namely oleoylcholine, isokobusone, hemorphin-4, and glutamylalanine (Fig. 7d). These four metabolites were significantly downregulated in the AMI model. Furthermore, the use of CF or BBP separately and in combination significantly altered the expression of oleoylcholine, isokobusone, and hemorphin-4. Additionally, the combination of CF and BBP significantly restored glutamylalanine expression. Oleoylcholine is involved in acylcholine metabolism [82–84], which is related to the production of TMA and TMAO [85]. TMA and TMAO are closely associated with the risk of CVDs. Glutamylalanine levels are reduced in abnormal metabolites in patients with heart failure [86]. Isokobusone inhibits the expression of inflammatory mediators induced by bacterial lipopolysaccharides [87]. Hemorphin-4 can inhibit angiotensin-converting enzymes and reduce death rate in patients with AMI and improve prognosis [88, 89]. This indicates that BBP combined with CF treatment may improve AMI by regulating the four aforementioned metabolites.

Spearman correlation analysis was conducted to investigate the potential functional correlation among the four dominant gut bacteria and four crucial intestinal metabolites relevant to the combination treatment of CF and BBP (Fig. 7e). These findings suggest that *Lactobacillus* significantly contributes to increasing the levels of hemorphin-4 and glutamylalanine after treatment with CF and BBP. However, *Butyricimonas* negatively correlated with these gut bacteria levels. Moreover, *Bilophila* showed negative correlations with the levels of hemorphin-4, glutamylalanine, and oleoylcholine. In addition, *Helicobacter* showed negatively correlated with the levels of hemorphin-4 and oleoylcholine (Fig. 7e). These results suggest that *Butyricimonas*, *Bilophila*, and *Helicobacter* may worsen cardiac damage after AMI. Combined CF and BBP treatment can potentially improve AMI by increasing the relative abundance of *Lactobacillus* and decreasing the relative abundance of *Helicobacter*, *Bilophila*, and *Butyricimonas*, further increasing the levels of hemorphin-4, glutamylalanine, and oleoylcholine, which are negatively correlated with aforementioned harmful gut bacteria that exacerbate cardiac damage after AMI.

## Discussion

STDP, which contains BBP, exhibits significant anti-AMI and anti-heart failure effects; nevertheless, the efficacy and compatibility mechanisms of BBP in STDP are still unclear. The efficacy of Chinese medicine has been closely linked to the gut microbiota. Moreover, the gut microbiota and metabolites can serve as effective and non-invasive biomarkers of AMI [90–92]. Our study revealed that the combination of CF and BBP had synergistic anti-AMI effects, as observed from the perspectives of gut microbiota, intestinal metabolites, and gene expression.

Changes in the internal environment can either disturb or repair the gut microbiota, promoting or improving the development of AMI. The concept of the gut-heart axis provides new light on the mechanisms underlying AMI [93]. The gut barrier is pivotal in the interaction between the intestinal bacteria and the heart [94]. Since the gut microbiota and cardiac function are closely related, targeting the gut bacteria could help individuals with AMI improve their heart function. Studies have shown that *Lactobacillus* enhances gut barrier integrity while reducing oxidative stress, inflammation, and myocardial injury [95]. *Lactobacillus* can improve cardiac function by reducing inflammation and myocardial damage after AMI [72]. We found that *Lactobacillus* and *Lactobacillaceae* showed an upward trend after the administration of the combined BBP and CF in rats with AMI, suggesting that CF and BBP may synergistically restore the relative abundance of *Lactobacillus* to alleviate AMI injury. Our study also found a positive correlation between glutamylalanine and *Lactobacillus*, which is similar to the findings of Ma et al. [96]. Additionally, Alonso-Roman et al. demonstrated that glutamylalanine supports the growth of *Lactobacillus *in vitro [97]. Glutamylalanine and *Lactobacillus* were associated with cardiac function. Glutamylalanine levels are reduced in patients with heart failure [86], while *Lactobacillus* can improve cardiac function by influencing inflammation- and oxidative stress-related factors in myocardial infarction [72, 73]. This suggests that glutamylalanine and *Lactobacillus* may contribute to the amelioration of AMI damage through mutual promotion. In addition to the correlation between glutamylalanine and *Lactobacillus*, other studies have also demonstrated correlations between other dominant metabolites and key gut bacteria identified in our study. The relative abundances of *Lactobacillus* and plasma oleoylcholine have been found to increase in patients with non-alcoholic fatty liver disease after undergoing autologous fecal transplantation [98]. There is also evidence that the precursors of oleoylcholine, oleic acid, and choline can increase the abundance of *Lactobacillus* and enhance its survival in gastric juices [99]. Oleoylcholine levels are closely correlated with the abundance of *Lactobacillus* in the intestine. Based on our findings that the combined use of BBP and CF improves AMI injury by increasing oleoylcholine levels through *Lactobacillus*, it is possible that *Lactobacillus* and oleoylcholine synergistically improve AMI injury.

RNA sequencing and AMI disease network revealed that the combination treatment of CF and BBP significantly restored 901 unbalanced genes in the AMI disease network. In comparison, BBP or CF alone restored 437 or 804 unbalanced genes that were affected by AMI modeling. These results provide new evidence of the synergistic effects of CF and BBP. The genes that were specifically restored after the combined use of CF and BBP were significantly enriched in the GO terms circulatory system process, regulation of system process, response to oxygen levels, and response to extracellular stimulus. Notably, EDN1 is crucial in the circulatory system process, regulation of system process, and response to oxygen levels. Wang et al. identified *Edn1* as a key gene involved in AMI via network control capability [54]. *Edn1* gene is crucial for maintaining normal cardiac contractile function, regulating the levels of superoxide and matrix metalloproteinase-9, and ensuring adequate collagen in the myocardium to prevent overstretching [52]. *Edn1* also defined as a critical gene in non-obstructive coronary artery disease [100]. Similarly, GRB2 is a downstream protein in the transforming growth factor beta 1 stimulated myofibroblast transformation process in cardiac fibrosis and heart failure [101]. The microRNA-378a/GRB2 pathway can promote cardiac fibrosis via lncRNAs in the myocardial infarction model [102]. Our study confirmed that EDN1 expression levels were decreased after treatment with combined CF and BBP, providing evidence for the synergistic anti-AMI effects of BBP and CF.

While this study contributes valuable insights into the scientific understanding of BBP compatibility in STDP, it does have several limitations. First, we utilized 16S rRNA sequencing to investigate the alterations in gut microbiota in rats with AMI; however, the effects of gut bacteria modified by the combination of CF and BBP treatment on rats with AMI were not further analyzed. Future research should focus on validating the gut microbiota analysis results through fecal microbiota transplantation experiments. Additionally, the impact of metabolites on cardiac-related cells warrants further investigation. Second, the use of drug-containing serum can more accurately mimic in vivo conditions and facilitate the assessment of the pharmacological effects of TCM.

## Conclusion

In this research, we revealed the synergistic effects of combined BBP and CF in the AMI model from the perspectives of gut microbiota, intestinal metabolites, and gene expression. These studies demonstrated that treating with combined CF and BBP significantly recovered AMI compared with either treatment alone. The upregulation of *Lactobacillus* and downregulation of *Helicobacter*, *Bilophila*, and *Butyricimonas* could affect intestinal metabolites, potentially aiding AMI treatment. Moreover, combination treatment with BBP and CF may have a positive impact on the treatment of AMI by restoring the AMI disease network, yielding a gene reversion rate of 62.34%. These results provide valuable data to support the scientific understanding of the compatibility of BBP in STDP.

## Supplementary Information

Supplementary Material 1. Fig. S1. The combination effect of CF and BBP on serum CK, LDH and IL-6 levels. Fig. S2. Effects of combined administration of CF and BBP on the gut microbiota of rats with AMI based on 16S rRNA. Fig. S3. Volcano plots of differential metabolites analyzed by LC–MS and GC–MS. Table S1. Elution gradient parameters of LC. Table S2. Mass spectrometry parameters. Table S2. Mass spectrometry parameters.

## Data Availability

The datasets used and/or analyzed during the current study are available from the corresponding author on reasonable request.

## Abbreviations

AMI: Acute myocardial infarction
BBP: Bear bile powder
CCK-8: Cell counting kit-8
CF: STDP without BBP
CI: Combination index
CK: Creatine kinase
CVDs: Cardiovascular diseases
EDN1: Endothelin 1
EoR: Efficiency of recovery regulation
FPKM: Fragments per kilobase of transcript per million fragments mapped
GAPDH: Glyceraldehyde 3-phosphate dehydrogenase
GC–MS: Gas chromatography–mass spectrometry
GO: Gene ontology
GRB2: Growth factor receptor-bound protein 2
H&E: Hematoxylin and eosin
HUVECs: Human umbilical vein endothelial cells
IL-6: Interleukin-6
LC–MS: Liquid chromatography–mass spectrometry
LDA: Linear discriminant analysis
LDH: Lactate dehydrogenase
LefSe: Linear discriminant analysis effect size
LVEF: Left ventricular ejection fraction
LVFS: Left ventricular fractional shortening
LPS: Lipopolysaccharides
MTT: Methylthiazolyldiphenyl-tetrazolium bromide
NO: Nitric oxide
NTRA: Network topology and transcriptomics-based approach
OGD: Oxygen and glucose deprivation
PCoA: Principal coordinate analysis
PLS-DA: Partial least squares discriminant analysis
PPI: Protein–protein interactions
RT-qPCR: Real-time quantitative reverse transcription polymerase chain reaction
SEM: Standard error of the mean
STDP: Shexiang Tongxin dropping pills
TBST: Tris-buffered saline with Tween-20
TCMC: Traditional Chinese medicine compatibility
TCMs: Traditional Chinese medicines
TMA: Trimethylamine
TMAO: Trimethylamine N-oxide
TRPC6: Transient receptor potential cation channel subfamily C member 6
TTC: 2,3,5-Triphenyltetrazolium chloride
