# Supplementary material for: Efficacy and compatibility mechanism of bear bile powder in Shexiang Tongxin dropping pills for acute myocardial infarction treatment
Source: Chin Med. 2025 Jan 25;20:14. doi: 10.1186/s13020-025-01060-x (PMC11763157; doi:10.1186/s13020-025-01060-x)
Supplement: Supplementary file 1 — Supplementary Material 1. Fig. S1. The combination effect of CF and BBP on serum CK, LDH and IL-6 levels. Fig. S2. Effects of combined administration of CF and BBP on the gut microbiota of rats with AMI based on 16S rRNA. Fig. S3. Volcano plots of differential metabolites analyzed by LC–MS and GC–MS. Table S1. Elution gradient parameters of LC. Table S2. Mass spectrometry parameters. Table S2. Mass spectrometry parameters. [file 13020_2025_1060_MOESM1_ESM.docx]

**Supplementary Method**

**Enzyme-linked immunosorbent assay (ELISA)**

To detect inflammation levels in rats with AMI, we measured serum interleukin-6 (IL-6) concentrations. Before analysis, blood samples were centrifuged at 4,000 rpm for 10 minutes to collect serum. The levels of IL-6 in serum were measured by an ELISA kit (E-EL-R0015, Elabscience, Wuhan, China). The combination index (CI) was calculated using the methods and equations described in section 2.5 of the manuscript. The combined effect of two drugs can be quantified as$E_{CF+BBP}$ (0 ≤$E_{CF+BBP}$ ≤ 1), and when CI is less than 1, it implies that the two have a synergistic impact.

**Supplementary Figures**


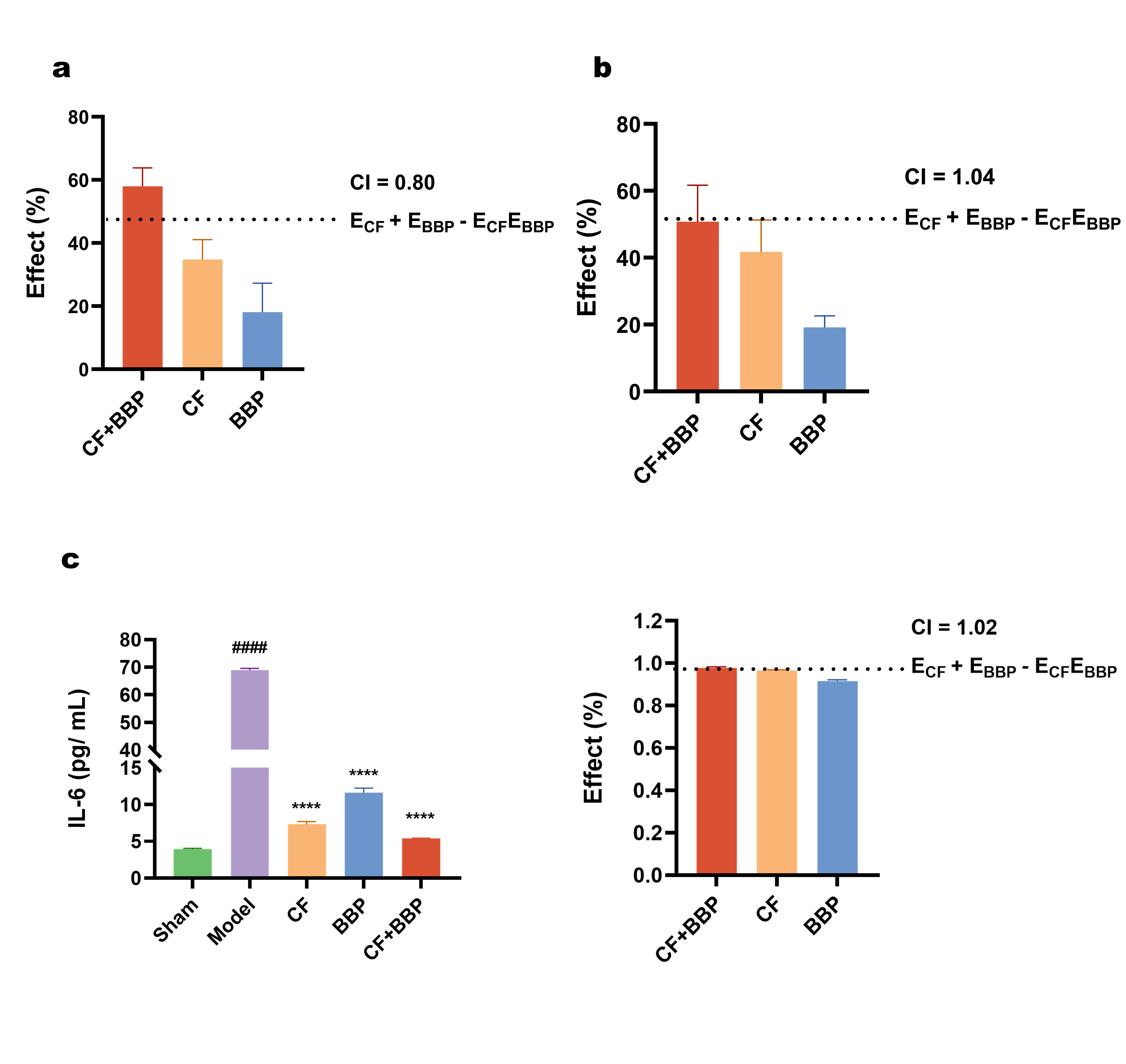


**Fig. S1.** The combination effect of CF and BBP on serum CK, LDH and IL-6 levels. (**a**) The CI of CF and BBP on the serum CK levels in rats with AMI. (**b**) The CI of CF and BBP on the serum LDH levels in rats with AMI. (**c**) Serum IL-6 levels and the CI of CF and BBP on it in rats with AMI, *n* = 5.


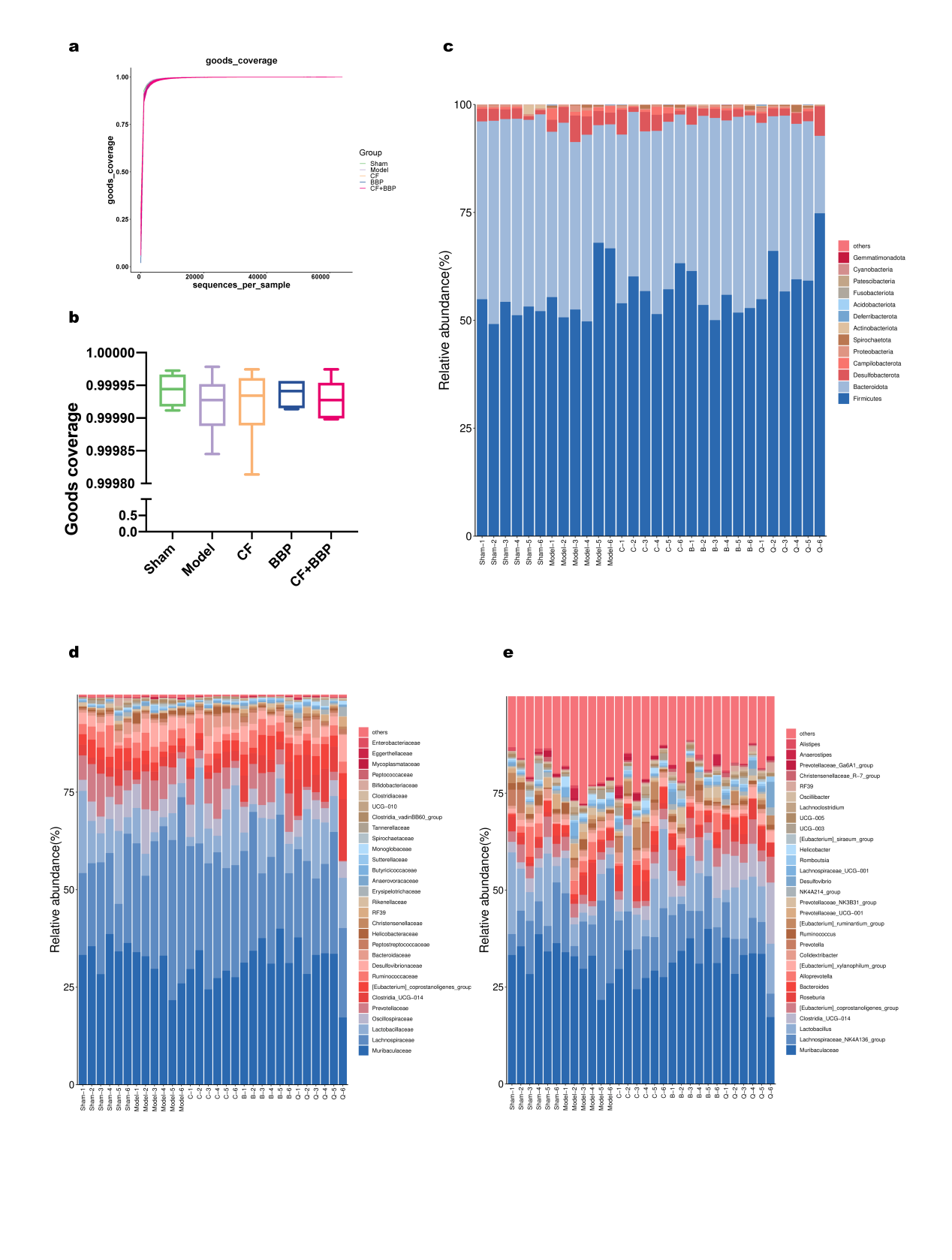


**Fig. S2.** Effects of combined administration of CF and BBP on the gut microbiota of AMI rats based on 16S rRNA. (**a**) Dilution curve based on Good’s Coverage index. (**b**) Alpha diversity comparison based on Good’s Coverage index. Gut microbiota composition of sham-operated rats or AMI rats treated with BBP, CF, combined BBP and CF or saline at the phylum (**c**), family (**d**), and genus (**e**) levels. *n* = 6 per group. CF, separated prescription (STDP without BBP); BBP, bear bile powder; BBP + CF, the combination of BBP and CF.


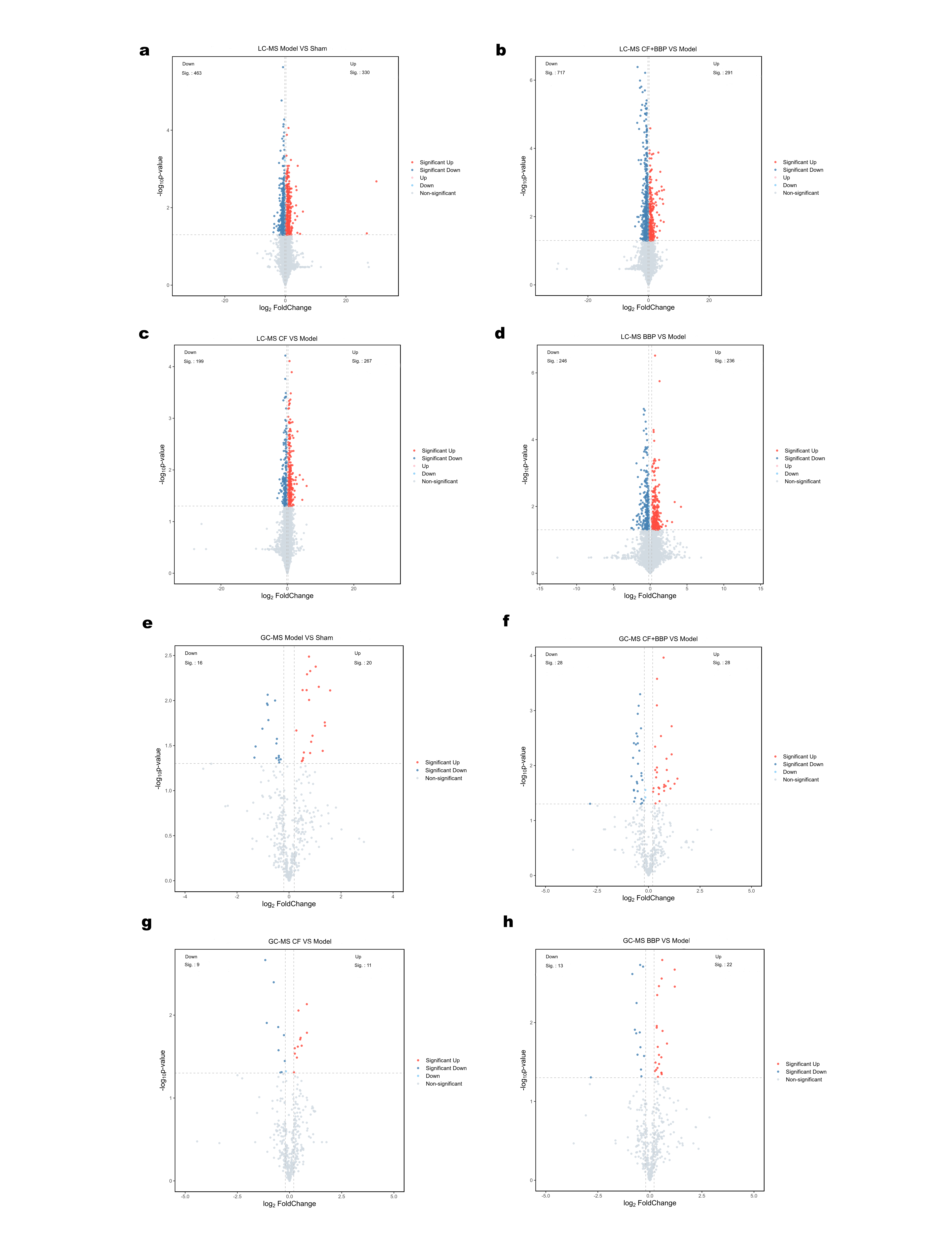


**Fig. S3.** Volcano plots of differential metabolites analyzed by LC–MS and GC–MS. Differential metabolites between Sham and Model groups (**a**), Model and CF + BBP groups (**b**), Model and CF groups (**c**), Model and BBP groups (**d**) based on LC–MS. Differential metabolites between Sham and Model groups (**e**), Model and CF + BBP groups (**f**), Model and CF groups (**g**), Model and BBP groups (**h**) based on GC–MS. *n* = 6 per group. CF, separated prescription (STDP without BBP); BBP, bear bile powder; BBP + CF, the combination of BBP and CF.

**Supplementary Tables**

**Table S1.** Elution gradient parameters of LC

| **Time (min）** | **Mobile Phase A%** | **Mobile Phase B%** |
| --- | --- | --- |
| 0-2 | 95 | 5 |
| 2-4 | 95-70 | 5-30 |
| 4-8 | 70-50 | 30-50 |
| 8-10 | 50-20 | 50-80 |
| 10-14 | 20-0 | 80-100 |
| 14-15 | 0 | 100 |
| 15-15.1 | 0-95 | 100-5 |
| 15.1-16 | 95 | 5 |

**Table S2.** Mass spectrometry parameters

| **Parameters** | **Positive ions** | **Negative ions** |
| --- | --- | --- |
| Spray voltage（V） | 3800 | -3000 |
| Capillary temperature（℃） | 320 | 320 |
| Aux gas heater temperature | 350 | 350 |
| Sheath gas flow rate（Arb） | 35 | 35 |
| Aux gas flow rate（Arb） | 8 | 8 |
| S-lens RF level | 50 | 50 |
| Mass range（m/z） | 100-1200 | 100-1200 |
| Full ms resolution | 70000 | 70000 |
| MS/MS resolution | 17500 | 17500 |
| NCE/stepped NCE | 10, 20, 40 | 10, 20, 40 |

**Table S3.** Sequences of primers for RT-qPCR

| **Gene** | **Forward Primer（5'- 3'）** | **Reverse Primer（5'- 3'）** |
| --- | --- | --- |
| *Edn1* | TCCAAGAGAGGTTGAGGTGTTCCC | CCAGACAGCAAGAAGAGGCAAGAG |
| *Grb2* | CGTGTCCAGGAACCAGCAGATATTC | AATGAAGTCTCCTCGGCGAAAGC |
| *Trpc6* | GCGGCAGACAGTTCTTCGTGAG | GCATCCAGAAAGCGTTCCTCCTC |
| *Runx2* | CTTCGTCAGCGTCCTATCAGTTCC | TCCATCAGCGTCAACACCATCATTC |
| *Comt* | ACCCCTGACTTCCTGGCGTATG | GCCTTCTCCAAGCCGTCTACAAC |
| *Hamp* | AAGGCAAGATGGCACTAAGCACTC | GCCGTAGTCTGTCTCGTCTGTTG |
| *Fads1* | TTGTGTGGGTGACGCAGATGAAC | AGTTGTTGAAGGCTGACTGGTGAAC |
| *Gapdh* | ACGGCAAGTTCAACGGCACAG | CGACATACTCAGCACCAGCATCAC |
